# State of play of CME in Europe in 2015: Proceedings from the Eighth Annual European CME Forum

**DOI:** 10.3402/jecme.v5.32174

**Published:** 2016-06-10

**Authors:** Eugene Pozniak, Anne Jacobson

**Affiliations:** ^a^ Siyemi Learning, University of Manchester Innovation Centre, Manchester, UK; ^b^ CPD Content Group, Cocoa Beach, FL, USA

**Keywords:** CME, CPD, accreditation, Europe, providers, best practice, funding, regulation, evaluation, outcomes

## Abstract

European CME Forum is a not-for-profit organisation that brings together all stakeholder groups with an interest in European continuing medical education (CME) and promote multichannel discussion in an independent and neutral environment. This report summarises the discussions that took place at the 8th Annual European CME Forum in Manchester on 11–12 November 2015. Held at a time of increased scrutiny on the quality and value of the CME, the forum provided a space for attendees to share perspectives on trends, challenges, and opportunities related to European CME accreditation, funding, and regulation. Discussions focused on specific “hot topics” identified through a pre-meeting survey and needs assessment conducted among CME stakeholders in Europe and beyond. Chief among these were issues related to managing the transparency of relationships between industry and healthcare professionals, evolving systems of European CME accreditation, and the future of CME funding. The programme structure included multiple workshops conducted by leaders in the CME field, and plenary sessions that facilitated multidisciplinary interactions with invited guests, including the very learners the CME field is designed to serve. Attendee feedback was gathered to begin shaping the programme for the 9th Annual European CME Forum (#9ECF), which will take place in Amsterdam, The Netherlands, on 9–11 November 2016.

## Introduction

European CME Forum is a not-for-profit organisation that was established in 2007 in order to bring together all stakeholder groups with an interest in continuing medical education (CME) and continuing professional development (CPD) for healthcare professionals across Europe. The 8th Annual European CME Forum (#8ECF) was held in Manchester on 11–12 November 2015.

**Figure 1:**
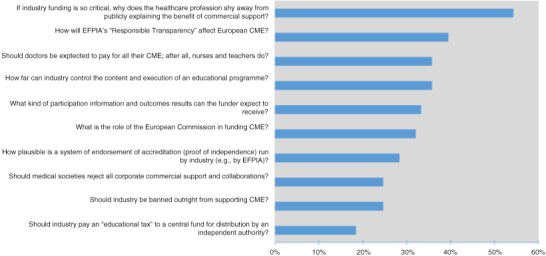
Responses to the pre-forum survey question, “What scenarios cause you the most concern?” (*N* = 88). CME, continuing medical education; EFPIA, European Federation of Pharmaceutical Industries and Associations.

To plan the meeting programme, the European CME Forum conducted a formal online needs assessment survey among CME stakeholders. With promotion by email and social media (LinkedIn and Twitter), the survey gathered responses from 88 individuals. Respondents ranked several scenarios as areas of the most concern for CME–CPD stakeholders (Figure 1). Survey respondents also identified the top three areas of interest for discussion at the forum: “measuring educational outcomes of CME activities,” “how CME can affect clinical practice,” and “industry's responsibilities when funding CME in Europe.” This feedback shaped the #8ECF agenda and proved prescient, as meeting attendees guided the discussion back to these topics throughout the 2-day meeting.

During the welcome session, Eugene Pozniak (Programme Director, European CME Forum, UK) opened the meeting by reviewing some of the major milestones within the CME–CPD community over the prior 12 months (Table 1).

**Table 1:** Major milestones in the CME–CPD community in 2015.

January	ACEhp Annual Meeting, Dallas^1^

May	gCMEp Spring Meeting, London^2^
June	EBAC Provider Meeting, Frankfurt^3^
July	Graham McMahon replaces Murray Kopelew as CEO of ACCME^4^
September	AMEE/GAME Joint Annual Meeting, Glasgow^5^
	4th Cologne Consensus Conference, Cologne^6^
	*JCEHP*/*JECME* joint editorial^7^
October	UEMS–EACCME holds Board elections including Secretary General position^8^
November	International Academy of CPD Accreditors, gCMEp, iPACME meet during “Day 0” at #8ECF
	8th Annual European CME Forum, Manchester

#8ECF, Eighth Annual European CME Forum; ACCME, Accreditation Council for Continuing Medical Education; ACEhp, Alliance for Continuing Education in the Health Professions; AMEE, Association for Medical Education in Europe; CEO, chief executive officer; CME, continuing medical education; CPD, continuing professional development; EBAC, European Board for Accreditation in Cardiology; GAME, Global Alliance for Medical Education; gCMEp, Good CME Practice group; iPACME, International Pharmaceutical Alliance for CME; *JCEHP*, *Journal of Continuing Education in the Health Professions*; *JECME*, *Journal of European Continuing Medical Education*; UEMS–EACCME, European Union of Medical Specialists–European Accreditation Council for Continuing Medical Education.

### Journal of European CME

The *Journal of European CME* (*JECME*), led by Editor-in-Chief Robin Stevenson and published by Co-Action, is an online-only Diamond Open-Access journal focused on CME–CPD practice in Europe and the global community at large. As well as being free to access, *JECME* is currently free for authors to submit to, thanks to additional financial support from the European Board for Accreditation in Cardiology (EBAC) and European Union of Medical Specialists (UEMS). *JECME* has seen steady growth in the number of original manuscripts published each year: two each in 2013 and 2014; six in 2015; and eight in the first quarter of 2016. In September 2015, *JECME* announced a collaboration with the *Journal of Continuing Education in the Health Professions* (*JCEHP*) to strengthen links between the two journals via shared announcements and joint editorials.^7^

*JECME* is already indexed/listed on Google Scholar, ResearchGate, and other databases, and has launched “Project PubMed” to finalise the process of being indexed on PubMed. After publishing eight more articles in 2016, *JECME* will reach this crucial milestone. The journal is accepting manuscript submissions, with a target time-to-publication of 6–8 weeks. Delegates were urged to consider submitting a publication to contribute to the ongoing discussion of best practices in European CME–CPD: www.jecme.eu.

### Good CME Practice group

The Good CME Practice Group (gCMEp; goodcmepractice.eu) was launched in 2009 as a membership organisation for European CME–CPD providers. In 2012, the gCMEp published best practices around four core principles in CME–CPD: appropriate education, effective education, fair balance, and transparency.^9^ Drawing upon the expertise within its current membership of 18 European CME–CPD providers, the gCMEp is developing a CME Toolkit to offer guidance on securing funding for CME-accredited activities, as well as detailed instructions on the effective delivery of these activities.

## Session 1: what is CME best practice today?

In the opening session of #8ECF, Alisa Pearlstone (PCM Scientific) and Sophie Wilson (International Medical Press), both members of the gCMEp, led delegates through interactive exercises designed to explore different perspectives on best practices in planning, developing, and implementing CME–CPD activities in Europe.

In the first small group activity, each table of delegates was asked to identify 20–25 essential elements for the delivery of a typical live European CME-accredited activity. Example steps included “perform a gap analysis,” “identify a potential supporter,” and “submit a grant.” Each step was written on an individual notecard to prepare for the next activity: CME Bingo. As the session leaders called out steps, members turned over their corresponding cards (if present) and raced to be the first to shout “Bingo!” The exercise revealed substantial diversity among meeting delegates in the degree of familiarity with what developing a CME activity entails. Some tables skipped major fundamental steps entirely (e.g. “secure funding”), while others went into extensive detail.

**Figure 2:**
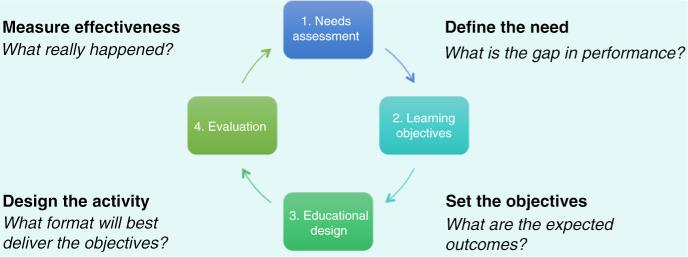
Educational design cycle (Pearlstone and Wilson).

During the next exercise, attendees were asked to review their notecards, identify the first six steps involved in developing a CME activity, and place those steps in sequence to reflect “normal practice” in the delivery of CME. Through this exercise, attendees discovered that there is no consensus that defines normal practice in CME design and delivery. Multiple approaches will lead to the desired endpoint, and many steps may happen in parallel rather than sequentially. Finally, delegates shared their experiences with the educational design cycle (Figure 2), which provides a framework for the continuous process of needs assessment and outcomes measurement.

## Sessions 2–4: forum workshops

New for this year was the forum workshop structure. Participants were able to choose which forum workshop they attended from a wide variety of contributors led by key organisations and individuals from the CME community. Nine workshops were presented during three sessions, divided into the following streams: Standards & Accreditation; Education & Partnerships; and Funding & Practice.

### Standards & accreditation

#### How to submit a CME application

Three years after launching new accreditation criteria for live educational events (LEEs), the UEMS–European Accreditation Council for Continuing Medical Education (EACCME) (www.eaccme.eu) realises that some aspects of the new criteria remain unclear to some providers. Edwin Borman (UEMS–EACCME) and Nathalie Paulus (UEMS–EACCME) reviewed the new UEMS–EACCME criteria, with a focus on areas that CME providers have found challenging to interpret.

The UEMS established the EACCME in October 1999. The UEMS–EACCME began accrediting LEEs in 2000 and e-learning materials in 2009. At present, the UEMS–EACCME accredits CME–CPD events taking place worldwide except for the United States and Canada. However, UEMS–EACCME has signed agreements of mutual recognition of credits with the American Medical Association (AMA) (for live events and e-learning) and the Royal College of Physicians and Surgeons of Canada (RCPSC) (for live events only). To date, the UEMS–EACCME has approved more than 2,000 applications for accreditation. Although 95% of applications are ultimately approved, this includes many cases in which UEMS–EACCME worked with providers to revise applications to get them to an approvable state. Approximately, 5% of applications are rejected.

The UEMS–EACCME issued new accreditation criteria in 2012 (UEMS 2012/30), effective from January 2013, for LEEs that highlight three key elements: greater emphasis on the needs of learners and the achievement of meaningful educational outcomes; increased transparency with regard to funding and organisation of LEEs; and high-quality education by the providers of LEEs. Consistent with these goals, the new criteria address four fundamental aspects of programme planning: funding; presentation of the programme book; conflict of interest (COI) and its resolution; and needs assessment, learning needs, and educational outcomes.

*Funding*. According to the updated accreditation criteria, the UEMS–EACCME will only consider for accreditation LEEs that fulfil specific requirements related to their funding (UEMS 2012/30-29). Funding can occur via the provider's own funds; registration fees; “unrestricted” or according to more compliant terminology, independent educational grants; exhibition booths; satellite symposia not recognised for CME; or a combination of these sources. Indeed, funding via independent educational grants from several industry sources is recommended.

Regardless of funding source, industry cannot be directly involved in the provision of the event. Therefore, as Borman explained, industry cannot invite participants and speakers or cover travel and accommodation costs of participants and speakers, although as a delegate questioned, this is not completely clear in the documentation provided by the UEMS. In addition, industry is not permitted to take part in the organisation of the event (e.g. registration of participants, event costs, staffing, catering, speakers’ fees, etc.) or in the development of the scientific programme (e.g. funding-company member on scientific or organising committee or on scientific programme, influence on choice of speakers, etc.). Events provided by the pharmaceutical and medical equipment industries will not be considered for accreditation. Several real-world examples from applications for UEMS–EACCME accreditation illustrate the acceptability and lack thereof of various funding sources (Table 2).

**Table 2:** Acceptability of hypothetical funding scenarios for live educational events (Borman and Paulus).

Source(s) of funding	Acceptability	Explanation

A medical device company supports hands-on training by :• Providing instrumentation • No other funding is listed	Not acceptable	A range of other techniques/tools should be made available to the learners. The education provided might be very good, but the UEMS–EACCME will not accredit this type of event as it is felt as being promotional (i.e. promotes the use of a particular product)
Activity is funded by: • Admittance fee • Income from rent of information booths to pharmaceutical companies and other interested parties • Unrestricted educational grant received from the event's sponsor	Acceptable	This is an example of multiple sources of appropriate funding
A single pharmaceutical company provides funding for: • Event costs • Meeting location, technical equipment • Speakers’ fees • Travel costs for speakers and participants • Accommodation for speakers and participants • Food and beverage for speakers and participants	Not acceptable	This programme includes a cornucopia of funding examples that violate the UEMS–EACCME accreditation criteria, including funding event costs, speaker fees, and travel costs and accommodation for participants

UEMS–EACCME, European Union of Medical Specialists–European Accreditation Council for Continuing Medical Education.

The UEMS–EACCME also provides guidance on which funding details should be provided in the application (UEMS 2012/30-30). The source(s) of all funding for the LEE must be declared and be made available to learners in a readily accessible manner. Failure by a provider to disclose the means of funding of an LEE will lead to rejection of its application. The provider must submit documentation confirming the basis of the funding for the LEE, whether this is by sponsorship, educational grant, or any other means. While all sources of funding must be declared, the actual amounts provided need not be.

In addition, all educational materials must be free of any form of advertising and any form of bias. The EACCME will reject any application that, in its opinion, includes advertising of any product or company directly related to any educational material (UEMS 2012/30-33).

*Presentation of a programme book*. The 2013 UEMS–EACCME accreditation criteria include updated guidance on the presentation of the programme book accompanying LEEs, which is especially relevant for the larger medical society congresses. The programme book should be divided into two parts. The first section must contain the scientific and educational information, including materials such as president's foreword; invitation; scope of the event; scientific/organising committees; list of faculty; programme overview; and scientific programme. Within the scientific programme and overview, if there are sponsored symposia they must be clearly described as “industry-sponsored symposium” but with no sponsor's name/logo and no further details.

The second section of the programme book should contain information about industry and other useful information about the event. This may include registration information and venue; social programme; acknowledgment of sponsors; listing/map of exhibitors; and list of sponsored sessions (with sponsor's name/logo and session details). Advertisements may be printed on the inside and outside back cover pages, but not on the inside front cover page.

Specific examples that will lead to automatic rejection of an application for accreditation include:

- The use of a sponsor's name in the title of the scientific programme, a scientific session, or a scientific lecture
- The display of brand names and/or individual company logos in scientific lectures or in the scientific programme
- The sponsor's name or logo on front cover of the programme book

*Declaration and resolution of COI*. The UEMS–EACCME criteria address COI reporting requirements for the Scientific and/or Organising Committee (UEMS 2012/30-24) and the programme faculty (UEMS 2012/30-27). Firstly, the provider must ensure that all members of the Scientific and/or Organising Committee provide written declarations of potential or actual conflicts of interest. Under current criteria, the written declarations must be provided upon submission of the application; however, this stipulation is under consideration by the EACCME Governing Board. Borman stated that the declarations must cover potential or actual conflicts of interest for the last 5 years and must indicate whether any fee, honorarium, or arrangement for reimbursement of expenses in relation to the LEE has been provided.

Secondly, he also explained that the provider must ensure that all members of the faculty provide written declarations of potential or actual conflicts of interest. Although these declarations do not need to be submitted with the application for accreditation, they must be available for potential review by the EACCME and must be retained for at least 1 year after the event.

Resolution of COI is also addressed (UEMS 2012/30-27). The provider must confirm that any actual conflicts of interest have been resolved by the following means:

- Declaration of COI as second slide of speaker's presentation
- Declaration of COI printed in scientific programme
- Declaration of COI published on event website

Evaluation forms completed by participants should include questions on the perception of speaker bias and invite comments on individual presentations. In the presence of actual COI, affected members of the Scientific and/or Organising Committee or faculty speakers should be excluded from participation.

*Needs assessment*. The provider must structure the LEE to fulfil defined educational needs (UEMS 2012/30-11). The application for accreditation must demonstrate that a needs assessment process has been completed, describe how that process was performed, and describe what relevant educational needs have been identified from that process. At minimum, needs assessments should identify the gap between current and desired clinical practise; be performed prior to developing a CME–CPD activity; and focus on learners’ knowledge, skills, and/or behaviours or patient or population outcomes or health. Acceptable sources for needs assessments include:

- Evaluation results from a previous activity
- Surveys of potential participants
- Publication of a new clinical guideline
- Publication of peer-reviewed research
- Publication of comparisons of clinical outcomes
- Population health data at regional/national/international level
- Legislative/regulatory/organisational changes affecting patient care
- Epidemiological data

Again, participants explored multiple real-world examples of needs assessments submitted to UEMS–EACCME to illustrate the threshold of acceptability (Table 3).

**Table 3:** Acceptability of educational needs for live educational events (Borman and Paulus).

Educational needs/expected outcomes	Acceptability	Explanation

Educational needs: • The faculty will use both video and PowerPoint presentations • Edited videos that only concentrate on the most interesting parts of the procedure will be used • The presenter will be available for questions and direct answers	Not acceptable	This is a statement of what is going to happen. This is NOT a needs assessment
Educational needs: • Independent patrons review and document the quality of the courses on a regular basis by means of consistent course-assessment procedures. The [provider's] scientific advisory board ensures an ideal selection of subjects and lecturers, according to the curricular activities of the societies. This board is made up of experienced medical practitioners from various disciplines and representatives from the area of nursing and hospital management	Almost acceptable	A needs assessment should identify what the target audience needs to learn. Although the proposed activity content is based on expert-level informed evidence, there are no educational needs stated
Educational needs: • Spasticity in children: causes, consequences • Cerebral palsy: topographical and severity classifications • Why spasticity is different in children: the role of plasticity and growth • Multidisciplinary management: pharmacological, orthopaedic, functional and surgical. When and how to treat? How to set goals? • Assessment of standard situations and development of a treatment plan • The role of botulinum toxin: effectiveness and safety • Guidelines on injections: who, what, when, doses for treatment, follow-up • Assessment: functional and spasticity scales • Practical considerations for botulinum toxin treatment: electromyography and electrostimulation-guided injections • Long-term follow-up	Not acceptable	This statement includes substantial information, and potentially an activity outline, but no assessment of needs
Educational needs: • Increased awareness of alcohol dependence as a treatable brain disease • Alternative treatment approaches • Familiarity with evidence for alcohol reduction and “as needed” dosing treatment approaches	Not acceptable	This statement offers an impression of what the provider wants people to learn, rather than a learner-based needs assessment
Participants will be able to: • Discuss unmet needs • Describe benefits of patient-centred management • Illustrate with clinical evidence the feasibility of reduction as an achievable treatment goal, and “as needed” dosing, in alcohol-dependent patients with a high drinking risk level	Almost acceptable	The learning objectives are too general. This statement needs to be more specific about identified educational needs
After attending this course, learners will acquire the skills to: • Recognise the value of a holistic approach to the management of food allergy in adults and children • Ensure they are aware of the most recent advances in the diagnosis and management of food allergy and non-allergic related adverse reactions to food • Perform oral provocation challenge • Understand the role of immunotherapy and initiate treatment safely	Acceptable	This statement provides an overview of what will be achieved, including specific knowledge and practice gaps that will be addressed

Providers regularly contact the UEMS–EACCME office requesting clarification on how to interpret the new accreditation criteria. The UEMS Governance Board on CME–CPD is always pleased to receive for consideration any suggestions from participants, providers, reviewers, and national accreditation authorities for further improvements.

#### Getting from gaps/needs to changes in learners

In the United States, the Accreditation Council for Continuing Medical Education (ACCME) oversees the provider-based accreditation system. To share the US perspective on educational planning, Kate Regnier (ACCME) reviewed multiple sources and approaches to identifying learner gaps/needs; how educational needs might be addressed through different activity formats; and how educational effectiveness is evaluated in terms of measuring change.

Practice gaps are defined as the difference between healthcare processes or outcomes observed in practice and those potentially achievable on the basis of current professional knowledge. Practice gaps can extend to multiple aspects of care, competence, performance, and patient outcomes. The ACCME's requirements for educational planning and evaluation highlight the identification of professional practice gaps and educational needs as the cornerstones of effective continuing education. Importantly, educational needs are specific and contextual; what an individual learner needs may be different from what an interprofessional team needs. Understanding educational needs also helps the educator to choose appropriate tools (and methods) to most effectively produce and evaluate change.

Current ACCME requirements reward accredited providers that take a strategic approach to maximize the effectiveness of their educational programmes to support change and improvement. After weighing the merits of different educational interventions, ACCME-accredited providers are expected to choose activity formats appropriate to the goals/objectives of the educational intervention and analyse the effectiveness of their efforts to produce changes in learners’ competence, performance, and/or patient outcomes. In this way, effective continuing education begins with the end in mind. To address problems in practice, one must understand what issues underlie the “gap” to determine what needs to be changed and improved. Workshop attendees considered several examples of statements of professional practice gaps, activity formats, and evaluation mechanisms to illustrate how these components can be implemented in the development of CME–CPD (Table 4).

**Table 4:** Sample provider statements on professional practice gaps, activity formats, and evaluation mechanisms (Regnier).

Provider statement	Consideration	Explanation

The field of transplant surgery is constantly evolving at a rapid pace and the healthcare team **needs to keep pace**. Annual updates on the **latest surgical techniques, patient selection, and treatment** for post-transplant care are needed	Has this provider identified a professional practice gap?	Yes. The bolded text supports a professional practice gap
Healthcare professionals rarely receive instruction on the skills needed **to be an effective educator** while in training. Our faculty have expressed a desire to learn **how to effectively provide feedback** to their learners	Has this provider identified a professional practice gap?	Yes. CME can be about “being an effective educator.” It does not need to be limited to clinical care problems
After participating in this “Train the Trainer” activity, our staff – who serve as faculty for all regularly scheduled series – will know **how to implement effective strategies** for giving feedback during educational events	Has this provider designed this activity to close a gap in the skill, strategy, performance of its learners?	Yes. This provider has designed this activity to close a gap in the skill, strategy, and performance of learners
“The upcoming ‘Train the Trainer’ activity will use **case scenarios and role playing** to achieve the goal of teaching our faculty **effective strategies for giving feedback** during educational events”	Has this provider chosen a format for the activity that will help achieve the expected results?	Yes. This provider chose a format for the activity that will help achieve the expected results
The provider will distribute an evaluation survey at the conclusion of the activity to ask how well the learners liked the content, format, and trainer	Will this evaluation mechanism help determine if the professional practice gaps have been closed?	No. This evaluation mechanism will not help determine if the professional practice gaps have been closed
The provider will ask that the faculty trainer use a standardised score card to **rate the learners on their feedback techniques** during role play	Will this evaluation mechanism help determine if the professional practice gaps have been closed?	Yes. This evaluation mechanism will help determine if the professional practice gaps have been closed

#### Global accreditation requirements and evolving standards

Jennifer Gordon of RCPSC provided an overview of trends, challenges, and opportunities in global CPD accreditation requirements. First, it is important to define basic terminology, as “accreditation” may hold different connotations across the spectrum of CPD stakeholders. At its most fundamental, accredited programmes are those that have been recognised for meeting minimum standards. Accreditation is also a process for facilitating quality improvement in education, as well as enterprise in which programme characteristics are evaluated against standards set by a third-party organisation.

Over the past several years, multiple international groups of experts have been bridging national boundaries and building common group across divergent accreditation systems around the world. Global CPD accreditation reached a major milestone in 2010, when stakeholders participating in the Second International Forum on CPD Accreditation in Sydney, Australia, developed the Sydney Consensus Statement that defines the values, principles, and metrics of CPD accreditation systems (Table 5). In 2013, the International Academy for CPD Accreditation was created to bring experts in CPD/CME accreditation together in one community.

**Table 5:** 2010 Sydney Consensus Statement on CPD Accreditation Systems (Gordon).


*Values*	*Principles*
CPD Accreditation systems should: (1) Be based on a reasonable set of standards and criteria for organisations or programs (2) Demonstrate accountability and fairness by defining and monitoring adherence to established standards and criteria (3) Promote continuous quality improvement of accreditation standards and processes (4) Encourage and foster effective collaboration and partnership with provider organisations (5) Value physician learning across a range of competencies relevant to professional practice (6) Promote strategies to improve physician performance and thereby improve the health of people Individual CPD events should: (1) Be based on information that defines the professional educational needs of physicians (2) Link assessment of needs to effective educational strategies (3) Evaluate the achievement of defined outcomes (4) Be developed without influence from commercial interests Individual physicians should: (1) Participate in CPD as a personal and professional responsibility (2) Identify their own needs (3) Participate in learning activities linked to their professional roles and responsibilities	Physicians are responsible to: (1) Participate in CPD is a personal and professional responsibility (2) Engaging in learning activities based on an assessment of professional needs derived from self-evaluation and external sources (3) Evaluate the impact of learning for their professional practice CPD organisations are responsible to: (1) Promote continuous learning in practice (2) Enhance self-directed learning as both a process and goal of professional education (3) Facilitate the translation of learning into practice (4) Enable or promote learning within communities or health teams *Metrics* Individual CPD activities should be designed to enhance one or more of the following:: (1) Knowledge of physicians (2) Skills and/or competencies of physicians (3) Performance of physicians (4) Performance of health teams (5) Health outcomes of patients

The International Academy for CPD Accreditation (academy4cpd-accreditation.org) is dedicated to promoting and enhancing the development, implementation, and evolution of CME–CPD accreditation systems throughout the world. The Academy provides opportunities for individuals in leadership positions within CPD to learn about the values, principles, and metrics of varying CME–CPD systems; explore the accreditation standards for CME–CPD provider organisations and activities under differing systems; and foster evaluations to measure the impact of CME–CPD accreditation systems on physician learning, competence, performance, and healthcare outcomes.

Several recent trends in global accreditation standards have emerged. First and foremost, given the increased focus among CME–CPD providers on interdisciplinary and interprofessional education (IPE), accreditation systems are exploring new options for joint accreditation (e.g. physician, nursing, pharmacist, etc.). Some systems are already providing team-based credits, while others recognise only individual learning within the IPE context. Another change in accreditation standards involves the shift beyond self-reporting, instead requiring learners to demonstrate accountability and improvement in performance. Furthermore, although education is often anchored in the learner's current roles and scope of practice, it is increasingly recognised that CPD should encompass a range of competencies that reflect the variety of practice settings learners may have throughout their career.

The analysis of global CPD trends also reveals several ongoing challenges. Accreditation standards around web-based learning and e-learning activities vary considerably worldwide. Although the 2004 ACCME Standards for Commercial Support marked an important milestone in defining commercial independence in CME, other international organisations have maintained their own criteria, leading to some variation in the degree to which commercial industry can be involved in educational programmes (e.g. development, sponsorship, blended models). COI standards also vary in terms of declaration requirements (financial relationship only vs. all relevant relationships), resolution requirements (disclosure only vs. full COI management strategies), and COI rating/weighting schemes.

Healthcare providers are increasingly seeking educational opportunities that are created and delivered globally. Learners often need these activities to be accredited or certified, yet individual CPD system requirements and standards continue to vary across the world. To bridge this gap, some accreditation systems are beginning to recognise other systems as “substantively equivalent.” Importantly, accreditation systems do not need to be identical to be deemed substantively equivalent. Systems can reach formal agreements based on consensus around key educational characteristics of CPD activities (e.g. 2010 Sydney Consensus Statement criteria) that overcome other differences in organisational and CPD system components in their respective countries. For instance, the AMA, UEMS, and RCPSC have established substantive equivalency agreements to facilitate learning across borders and encourage lifelong learning mobility for international healthcare providers. Physicians who attend accredited or certified CPD activities can claim or convert credits for recognition within their home systems.

As global CPD accreditation standards continue to evolve, the International Academy for CPD Accreditation has identified several key priorities for CPD stakeholders. These include:

- Developing an international CPD accreditation database
- Developing a taxonomy of CME–CPD accreditation systems
- Defining national standards for commercial support
- Supporting the harmonisation of CME–CPD accreditation systems

### Education & partnerships

#### Using a combined model for planning educational activities for interprofessional clinical teams

Ideally, educational activities are designed to address the identified learning needs of the specific target audience and facilitate the transfer of new knowledge into practice. How does this process change when the target audience is an interprofessional team? Don Moore (Vanderbilt University), Kathy Chappell (American Nurses Credentialing Center), Lawrence Sherman (TOPEC Global), and Mathena Pavan (University College London Hospital) shared best practices for planning effective educational activities for clinicians and interprofessional teams.

One effective tool for CME providers involves backwards planning, the method of designing educational activities by setting goals before choosing forms of assessment, and instructional methods.^10^ Backwards planning occurs in three key stages: identify the desired results; determine acceptable levels of evidence that demonstrate that the desired results have occurred (i.e. plan for assessment); and design activities that will make the desired results happen (i.e. plan for learning).

**Figure 3:**
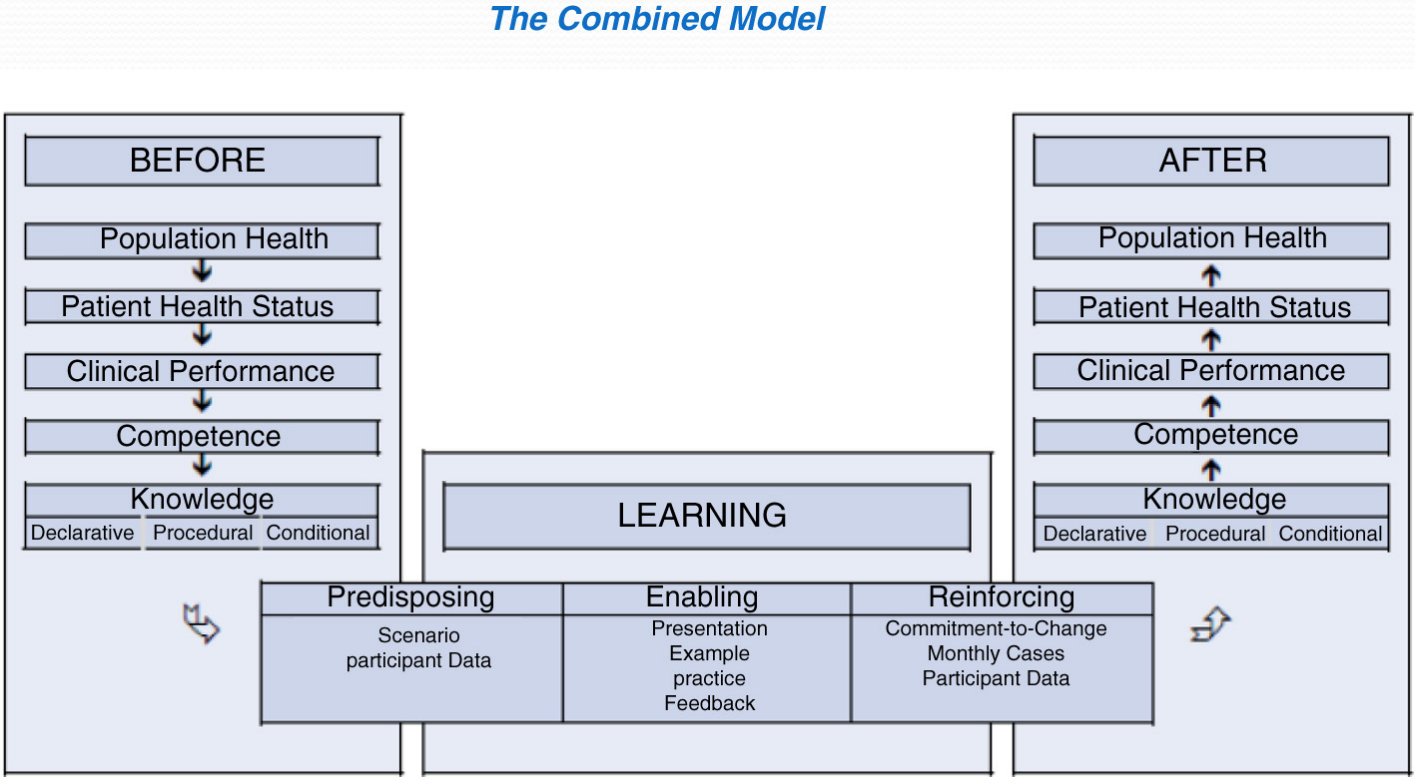
Combining the outcomes framework and instructional design models (Moore).

The CME Outcomes Framework is another theory-based approach to planning and assessing educational activities for physicians, nurses, and other health professionals as individuals and as teams.^11^ The seven-level framework progresses through participation, satisfaction, learning, competence, performance, patient health, and community health to help CME planners identify, plan for, and assess desired results. This framework is appropriate for both formal and informal learning conditions. Since it was introduced in 2009, the outcomes component of the framework has received considerable attention, while the instructional design component has been almost totally neglected. To achieve the desired results, backwards planning and instructional design must be used together in a combined model (Figure 3). Furthermore, assessments should occur to determine what an individual clinician or clinical team knows, does, should know, and should do. During the workshop, participants used the combined model to plan a hypothetical educational activity for an interprofessional team.

#### Implementing IPE to improve collaboration and patient outcomes

Kathy Chappel (American Nurses Credentialing Center) and Jann Balmer (University of Virginia) led participants through a lively interactive faculty-development workshop on IPE. Chappel and Balmer were invited to address this topic, which is emerging in the USA and currently rarely formalised in Europe. Historically, continuing education in the health professions has been conducted in silos, leaving little opportunity for practicing clinicians to learn from, with, and about each other. Yet, it is critical that clinicians practice collaboratively in teams. In the USA, academic institutions are incorporating IPE within pre-licensure or pre-registration curriculum. There are healthcare educators who develop IPE for practicing clinicians and aim to incorporate strategies to plan education that improves team performance and/or patient/system outcomes.

In this workshop, the participants, playing the role of healthcare educators, analysed a clinical scenario through the perspective of one of seven randomly assigned roles: general practitioner, neurologist, pharmacist, registered nurse, psychologist, patient and patient's spouse. An interprofessional planning committee was created and tasked with learning from, with, and about each other while evaluating the hypothetical educational needs around new treatment options for multiple sclerosis. Participants identified practice gaps that could be addressed through IPE and explored the relationships and dynamics that can support or impede collaboration. As a result of this exercise, participants were able to recognise the value of including all members of the healthcare team in order to truly understand the breadth and depth of problems in practice. In addition, delegates gained an appreciation of the additional time and energy required to engage all members of the healthcare team in planning continuing education that balances all viewpoints. Furthermore, workshop attendees explored the benefits and challenges associated with incorporating non-clinicians such as the patient and patient's spouse in planning continuing education.

#### The patient voice … a “novel” way to improve healthcare outcomes

In this panel discussion, Laura Muttini (Educational Health Services), Bobbie Hough (Aintree Hospital Rheumatology Patient Advisory Group representative), and Emma Rogan (European MS Platform; EMSP) reviewed global trends in patient education, engagement, and empowerment, and the impact of these trends on healthcare outcomes. First, Muttini presented findings from recent surveys that evaluated funding for independent patient education among members of the Industry Alliance for Continuing Education (IACE, formerly the Pharmaceutical Alliance for CME–PACME), a group of individuals from leading US pharmaceutical and devices companies with an interest in CME. Among survey respondents who reported supporting patient education in 2013 (*n*=32) and 2014 (*n*=27), common activity types included education for healthcare professionals with tools and/or education for patients (50% in 2013; 56% in 2014); stand-alone patient education (50% in 2013; 48% in 2014); integrated patient and healthcare professional education combined (59% in 2013; 44% in 2014); and patient simulations for healthcare professionals (44% in 2013; 30% in 2014). Only a small portion of respondents reported that their departments did not support independent patient education (16% in 2013; 19% in 2014).

To gain further insight into patient education trends, the Global Alliance for Medical Education (GAME) recently conducted the Global Patient Engagement Survey. Among 49 respondents, 63% reported including patients in CME–CPD–CE programming. Respondents described a diverse mix of programme types, including stand-alone patient programmes (8%), integrated programmes with clinicians (14%), and both stand-alone and integrated programmes (41%). Among activities that incorporated patient education, common activity types included discussion panels, printed materials, point-of-care tools, web-based platforms, and mobile applications. Survey respondents reported measuring outcomes from patient education programming to demonstrate not only participation but also changes in patient behaviour. Several tools are being used to measure health-related behaviour change, including pre/post programme survey questions, electronic medical records, and chart reviews. Funding sources for patient education programs are diverse and include pharmaceutical company grants, charitable donations, healthcare systems, government grants, patient societies, professional medical societies, and self-funding. GAME survey respondents were mostly optimistic about funding trends. Nearly 40% of respondents indicated that they expect funding for patient education to increase in 2016. By comparison, approximately 10% reported that funding will likely stay the same in 2016, and 8% expected funding to decrease. Overall, results from the GAME Global Patient Engagement Survey indicate widespread adoption of patient engagement strategies in CPD programming.

Next, Bobbie Hough described his personal story of living with juvenile rheumatoid arthritis (RA), becoming an RA patient advocate, and working with the National Health Service (NHS) to help influence hospital/health system changes. In 2012, the Rheumatology Patient Group (RPG) was formed at the Aintree University Hospital in Liverpool, UK, to provide a framework for RA patient education and engagement. However, the group faced a range of bureaucratic challenges, lack of buy-in from staff, lack of training, and other obstacles. Based on this initial experience, the RPG has since regrouped to enter phase II of their project, applying their lessons learned and additional patient feedback and guidance to move the project forward. The RPG experience not only illustrates the real-world challenges of establishing a patient advocacy group but also highlights the opportunities associated with successful patient engagement.

Then, Emma Rogan shared her personal story as an MS patient and described several successful patient engagement initiatives sponsored by EMSP (www.emsp.org). The EMSP programmes demonstrate that patients can not only be educated, engaged, and empowered, but also that patient programming can improve health-related outcomes. EMSP also develops awareness programmes for healthcare professionals and members of the general public. Programme examples include MS Nurse Pro, an e-learning training curriculum for nurses; the Under Pressure Project, which is a photojournalistic initiative about living with MS in Europe; and Believe and Achieve, a programme to address young adults being in the workplace and living with MS. The workshop participants were very engaged and responsive to learning through the use of these personal patient experiences.

Panellists discussed additional examples of successful online patient engagement programmes in a range of disease states using the Ayogo (ayogo.com) and ProPatient (propatient.com) simulation and gamification platforms. These platforms incorporate proven techniques for patient education, engagement, and support, and capture outcomes to demonstrate improvement in patient behaviours.

In summary, patient education and engagement are essential for supporting the patient's voice in shared-decision-making that affects their own healthcare outcomes. Patients also serve an important role in educating healthcare professionals to improve patient–provider communication, which further improves patient care.

### Funding & practice

#### The Amazing Race: the power and impact of CME–CE–CPD on global health issues

With the ever-evolving European CME Forum formats, the #8ECF meeting seemed an ideal time to introduce an element of “gaming.” Jann Balmer, (University of Virginia), Laura Muttini (Educational Health Strategies), and Lisa Sullivan (GAME) facilitated the Amazing Race workshop, which began with an overview of GAME. Founded in 1995, GAME (game-cme.org) is a US-registered not-for-profit international membership organisation focused on advancing innovation and collaboration in CME–CPD worldwide, with a goal of improving patient care. The current GAME membership includes a diverse group of more than 130 CME–CE–CPD stakeholders representing academia, health systems, medical societies, accreditors, and industry.

As many of the European CME Forum participants are actively involved in international CME deployment, the game format proved an excellent concept to challenge thinking and encourage collaboration with other team members often not well known to each other. By highlighting how different regions and countries manage CME–CE–CPD, this workshop explored the power of education and its impact on global health issues and crises. During the workshop, 18 CPD professionals played a game based on the TV show “The Amazing Race”. After splitting into two teams, the teams were given a packet that contained clues to help them determine “where they were.” The European countries represented were diverse in their structure and disease issues. Once each team successfully determined which country they were working with, they received another packet of clues to determine “what health crisis” they needed to solve. These represented serious public health issues globally with information from WHO fact sheets.

The teams then went to work on developing an innovative education and change strategy to address the health crisis they were handed, taking into account each country's CME–CE–CPD requirements and other specifics related to the education of healthcare professionals in their country or region. Having only 40 minutes to devise a plan, each team then presented the other team (and of course the judges) a brief overview of their ideas. Both reports were deemed excellent, and as it was too close to differentiate a winner, the winners’ names were drawn from the hat to receive a complementary registration to the 2016 GAME Annual Conference, which will be held in Barcelona, Spain, on 27 August 2016.

#### Industry support of medical education in Europe: a right, an obligation, or a privilege?

Maureen Doyle-Scharff (Pfizer, International Pharmaceutical Alliance for CME–iPACME) facilitated a discussion designed to explore the challenges and opportunities for industry support and engagement in medical education, given the fluid, changing landscape of healthcare in Europe. Participants shared insights into a range of topics related to industry support, beginning with the fundamental question: Why does and why should industry fund CME–CPD? Is it merely filling a void in public funding, or are there other motivations? Participants noted that CME–CPD is an avenue for discussing off-label use of medications, and such discussion may increase awareness of and enrolment in clinical trials. In addition, other companies may use CME–CPD support to raise their profile as a trusted brand in a particular disease area (e.g. as a responsible oncology provider).

Delegates generally supported the development of pan-European regulations to provide clarity across countries and agencies. Every country in Europe, whether EU member or not, has its own laws and regulations pertaining to industry relationships, and every pharmaceutical company has its own interpretation of those laws and regulations. As a result, even seemingly simple terms such as “bias,” “conflict of interest,” and “education” are interpreted differently across countries and regions. Perspectives also differ on the question of “content review” versus “content approval,” and the pressures that shape these processes. Notably, pharmaceutical companies in many non-US countries are legally responsible for the accuracy of scientific material. Accordingly, these companies feel legally obligated to review all content pertaining to the use of their products, whether the education is intended to be independent or not.

Regarding trends in funding, some workshop participants expressed concerns that industry interest in supporting CME–CPD will erode in response to increased regulations and documentation requirements around transparency. Others had the opposite view, arguing that transparency is the safest space for supporters to operate, especially in an era of increasing regulation. Several other valuable points emerged from the discussion:

- Multisupported CME–CPD is generally perceived as a potential safeguard against the perception of bias. Depending on the disease state or market niche (e.g. orphan disease), however, multisupport may not be a realistic goal.
- Accreditation systems are widening their reach. The ACCME has opened up accreditation to providers globally,^12^ and the UEMS–EACCME has hinted that provider accreditation may be around the corner. At present, UEMS–EACCME is exploring the possibility of a two-tiered system with “preferred” providers who enjoy fast-tracked accreditation applications. Doyle-Scharff suggested that accrediting providers rather than individual events is the natural next step in the evolution of European CME–CPD.
- When asked if industry-facilitated medical education should be accredited for CME–CPD, 23% of workshop participants agreed that it should; 43% said that it should not; and 33% reported that it “depends” on the circumstances.
- Given the pervasiveness of non-independent medical education programming, many learners do not understand the meaning of independent medical education and how it differs from other offerings. This is a common theme that was repeated throughout the #8ECF meeting.
- Learners generally have little awareness of how the UEMS–EACCME credit system works. In countries where healthcare professionals’ salaries are tied to obtaining country-level credits, there may be little interest in EACCME-accredited events.

#### How to work compliantly with faculty and industry support

Compliance describes either the state of being in accordance with established guidelines or specifications, or the process of becoming so. Marian East (MedSense; gCMEp) and Diana van Brakel (Kenes; gCMEp) shared strategies for implementing compliant activities within the changing environment of European CME–CPD.

The spectre of corporate sponsorship of CME programmes, such as meetings or e-learning initiatives, continues to be examined in the media and within the industry, with headlines shouting about bias, promotion, and undue influence. Despite bad press, most pharmaceutical companies aim to provide grants for ethically transparent and relevant CME programmes. Stakeholders closely examine accreditation guidelines to make sure strict criteria are met. Larger pharmaceutical companies establish CME departments separately from any marketing activities to ensure that best practice is undertaken and any possibility of influence or bias is eradicated. For smaller companies, however, uncertainty and confusion about “doing CME correctly” may persist.

To explore the nuances of compliant behaviour, Marian East and Diana van Brakel shared three case scenarios illustrating some of the trickier questions that CME providers are often asked. When country rules and regulations clash with accrediting bodies, what takes priority? As industry aims to become more CME compliant, what about faculty members who have many years’ experience of the old days of CME? How can CME providers help all parties understand that good CME practice makes for good educational outcomes? Workshops attendees shared their approaches to addressing the common challenges in CME programme implementation.

## Session 5: in conversation with…

Meeting attendees were able to eavesdrop on lively discussions between pairs of leaders from CME or directly related organisations. Delegates offered additional questions and comments to help guide the dialogue.

### Devolution Manchester and regionally focused CME–CPD

In the first conversation, Robin Stevenson (Editor-in-Chief, *JECME*) and Ian Bruce (Professor of Rheumatology, University of Manchester) discussed the Devolution Manchester project (“DevoManc”), the new initiative wherein Greater Manchester takes on the devolved responsibility of managing its own NHS budget, healthcare, and social care provision. DevoManc covers an area of roughly 3.5 million residents, and the plan is expected to reach full devolution during 2016.

The transition from national to regional oversight represents an opportunity to redefine best practices in regionally focused CME–CPD programmes. However, one of the major challenges is the absence of local/regional CME–CPD infrastructure. Unlike in the United States, where all major academic medical centres have internal CME departments, formal mechanisms for delivering medical education in the UK end at the postgraduate level. As an example, Bruce observed that rheumatology consultants are challenged to keep pace with the increasing complexity of treatment options. Whereas rheumatologists had just a single class of biological agents available for treating RA only 5–10 years ago, the current National Institute for Health and Care Excellence (NICE) guidance on RA management addresses the roles of 10 biological agents.^13^, (14) Rheumatologists cannot differentiate the subtleties of these treatments without sophisticated case-based educational programmes.

Within the DevoManc pilot programme, better linkage of electronic health and social care networks will allow better measurement of patient outcomes, healthcare savings, and other endpoints. To illustrate the range of potential regional metrics that can be captured, Bruce described the example of a hypothetical trial evaluating a new insole that changes the functional dynamics of the knee in patients with knee osteoarthritis. Linked e-health and social care networks can measure in real time whether patients using the insole are reporting less pain, experiencing less disability, and requiring fewer knee replacement procedures, thereby reducing healthcare costs. The next step is sharing those findings with general practitioners, nurses, physiotherapists, and podiatrists – each with different roles in managing patients with knee osteoarthritis, different gaps, and different educational needs.

Within DevoManc, there is the potential for CME–CPD to be embraced as a tool for shaping local healthcare delivery. Even city council members are getting involved, with an interest in improving healthcare cost efficiency. New CME–CPD programmes will test whether local healthcare providers are willing to change their practice-related behaviours to address local practice gaps identified within their own patient populations. Should the DevoManc initiative prove successful in changing healthcare practice towards better patient outcomes, it may eventually serve as a framework for similar regional programmes throughout the UK and Europe.

### European accreditation systems

Next, Craig Campbell (RCPSC) and Reinhard Griebenow (European Cardiology Section Foundation, ECSF) discussed trends in European CME–CPD accreditation systems through the perspective of EBAC (www.ebac-cme.org). In 2000, EBAC was formed as a joint initiative between the European Society of Cardiology (ESC) and the UEMS Cardiology Section to accredit cardiology CME–CPD programmes for the European medical community. Between 2001 and 2008, EBAC accredited 1,179 live events and 225 enduring activities (e.g. e-learning modules and CME articles). In 2009, ESC took the decision to withdraw from the joint venture. At present, EBAC continues to work with the UEMS–EACCME under the umbrella of the ECSF to accredit educational programmes covering the full spectrum of cardiovascular medicine.

Accrediting CME–CPD programmes in Europe is complicated by the presence of 27 member states of the European Union (as well as the non-member countries), each with its own national system of healthcare regulations. Without involvement from the European Commission, there is no overriding legislation regarding CME–CPD in Europe in the relevant countries. Where national accreditation systems exist, the CME–CPD credits are valid only within those individual countries. In the absence of reciprocity agreements, international learners who travel to participate in live programmes accredited by the host country's national system will not be able to claim the CME–CPD credits at home. With the emergence of UEMS–EACCME, EBAC, and other European accreditation systems, the new challenge is to ensure that the international CME–CPD credits are recognised by multiple national systems. Certificates issued by EBAC are currently recognised across Europe, but recognition criteria within individual countries are a moving target.

The field of CME–CPD is under pressure from multiple stakeholders. With no single entity driving change, accreditors have an opportunity to shape the future of European CME–CPD. In particular, accreditors can take a leadership role in defining best practices around disclosure and management of COI.^15^

### EFPIA and the challenges of transparency

Lastly, Marie-Claire Pickaert (European Federation of Pharmaceutical Industries and Associations, EFPIA) and Maureen Doyle-Scharff (Pfizer) discussed trends in European CME–CPD funding and changes within industry regarding disclosure and transparency. EFPIA (www.efpia.eu) currently represents 33 national medical associations, 39 leading pharmaceutical companies, and more than 1,900 companies involved in drug research and development. In 2010, EFPIA launched a programme to define ethical standards around industry relationships with healthcare professionals. As part of that initiative, EFPIA established a mandatory disclosure policy that required adoption into national codes by the end of 2013. The EFPIA code imposes obligations for industry to disclose transfers of value from pharmaceutical companies to healthcare professionals and healthcare organisations, with reporting beginning in 2016 for transfers occurring in the 2015 calendar year.

Transitioning into an era of disclosure and transparency has been difficult worldwide. In the United States, the 2010 Physician Payments Sunshine Act (PPSA) was met with strong resistance from the CME–CPD provider community. Doyle-Scharff observed that providers struggled for years to interpret the PPSA mandates and to report appropriate data on payments or other transfers of value given to healthcare providers. At one point, Pickaert received more than 600 emails per week asking for clarification around the definitions of direct and indirect transfers of value. In addition, some companies initially attempted to escape reporting by hiding behind third-party vendors. However, after the initial period of resistance, EFPIA members now recognise the importance of complying with the self-regulation as a condition of membership.

Pickaert and Doyle-Scharff also discussed the pronounced differences between the USA and Europe regarding the role of pharmaceutical companies in educating healthcare professionals. EFPIA member companies have a history of involvement in the full spectrum of medical education, from branded promotional education to independent CME–CPD. Although the relationships are regulated, the pharmaceutical industry in Europe is considered a legitimate partner and direct source of information on treatment safety and efficacy for healthcare professionals. By comparison, members of the US Pharmaceutical Research and Manufacturers of America (PhRMA) have only recently started to re-evaluate their role in serving the greater healthcare system through independent medical education, and as a result have come to recognise the fundamental value of CME–CPD in closing practice gaps and improving patient outcomes. Accordingly, several US pharmaceutical companies are transforming their independent grants departments to focus on clinical practice gaps.

As the underlying goals of CME–CPD evolve, Pickaert believes that there is an ongoing need for diverse offerings in medical education, from branded education to independent CME–CPD. Multiple channels of knowledge transfer from industry to healthcare professional are necessary to support clinicians in making informed decisions about safe and effective patient care.

## Session 6: the Value of CME 1: CME practice today

Independent moderator Jacqui Thornton opened the first of two “Value of CME” sessions by exploring current challenges and best practices in CME–CPD. Vassilios Papalois (Secretary General-elect, UEMS), Mark Westwood (St Bartholomew's Hospital), and Alistair Thomson (Consultant Paediatrician; Associate Postgraduate Dean, Health Education NorthWest) discussed the practicalities of presenting relevant and appropriate education today.

### CME–CPD infrastructure

Whereas undergraduate and postgraduate medical education functions well for medical students and junior doctors, with specific educational objectives and a clear curriculum, the lack of an established infrastructure for CME–CPD hinders the quality of education at the consultant/specialist level. In the UK, the General Medical Council (GMC) recently instituted new standards for undergraduate through postgraduate medical education, including a “Train-the-Trainers” programme designed to support medical educators.^16^ Effective from 31 July 2016, trainers must be formally recognised by postgraduate deans and medical schools, and will “receive the support, resources, and time to meet their education and training responsibilities.” Ideally, the programme will influence trainers’ attitudes towards their own lifelong learning and the continuum of medical education.

### Medical congresses

Panellists engaged in a lively discussion of the educational value of medical conferences, particularly the “mega-congresses” where delegates can earn 20 or more external CME credits per meeting. How well can these meetings meet the diverse needs of today's learners? Participants discussed multiple tools for enhancing the educational value of society meetings: incorporating more interactive elements into otherwise didactic presentations; using case-based presentations to explore the nuances of clinical decision-making; mixing session formats throughout the day; and surveying the membership to identify learners’ needs. Smaller society meetings – including those representing new subspecialties with no established curriculum (e.g. cardio-oncology) – tend to present more innovative educational content.

### Annual appraisal system

The process of planning and reviewing each practitioner's personal profile of CME–CPD activity is flawed. Due to time constraints, the annual CME–CPD appraisal is often compressed into a single meeting and/or combined with a discussion of job planning, which is a separate consideration. Meeting attendees agreed that the culture around CME–CPD appraisal needs to change, with an increased emphasis on individualised learning needs and continuous re-evaluation of the learning plan.

### Uniform training standards

Panellists expressed concerns over several trends in specialist training. At present, the standards for specialist training and appraisal vary considerably across Europe. In addition, there is increasing pressure to make undergraduate and postgraduate medical education shorter and more general, which shifts the responsibility for specialist training into the realm of CME–CPD. Given the looming shortage of healthcare professionals, there is a fear that the standards of specialists training may be lowered further, with a greater reliance on allied health professionals. Together, these pressures form a strong rationale for establishing pan-European standards for the quality of specialist training. As CME–CPD plays a greater role in training specialists and subspecialists, European specialty societies can lead the development of a standardised training curriculum to safeguard against eroding quality.

### Funding

Given that fulfilling CME–CPD credit requirements is a mandatory condition of employment, the current model of self-funding can be considered burdensome and unfair. Countries, regions, and hospitals differ in the level of support they provide to trainees and consultants. In London, one hospital provides trainees with £800 per year to cover all educational needs. For consultants, who are considered “trained” and less in need of instruction, this is reduced to £600 per year, less than the registration fee for one major medical meeting. Furthermore, some hospitals offer few or no internal CME–CPD events, leaving it up to the clinician to find other educational opportunities that are supported by industry or self-funded. Although industry provides direct funding for healthcare professionals to cover meeting attendance, this practice puts clinicians in a vulnerable position by introducing the perception of COI. Panellists explored the idea of an industry-supported central fund, administered by hospitals or another third party to sponsor meeting attendance, but agreed that it would not be practical.

Strengthening internal hospital-based programmes, as well as local and regional CME–CPD offerings, is necessary to relieve the burden of self-funding on individual healthcare professionals. To summarise the merits of funding, Thomson shared a quotation attributed to Derek Bok, former president of Harvard University: “If you think education is expensive, try ignorance.”

## Session 6: the value of CME 2: lunch with the learners – what does our target audience think?

In a return of the most popular session format at European CME Forum, a panel of local learners, under the watchful eye of Lawrence Sherman (TOPEC Global), discussed and debated with the audience how they consume the education the CME–CPD community develops, presents, and accredits. Panellists included Ciara O'Brien (NHS Christie Trust), Benjamin Parker (Central Manchester University Hospitals, NHS Foundation Trust), John Reynolds (Central Manchester University Hospitals, NHS Foundation Trust), and Sarah Skeoch (University of Manchester).

### Medical congresses

Most panellists rely on medical congresses to meet the bulk of their annual CME–CPD credit requirements. Learners attend about two major meetings per year to earn most of their credits, and supplement their annual CME–CPD portfolios with local programmes and/or e-learning.

### Networking opportunities

Learners value the opportunity to network with colleagues at medical congresses, provided the interactions are not forced. One panellist described a successful networking format around a moderated poster discussion. In the session, investigators presented three to four posters, and congress delegates chatted in small groups about the research findings after each presentation. The network event was successful because participants were a self-selected group with shared interests on a specific topic, which provides a foundation for natural interaction. By comparison, the panellists described “speed networking” events among individuals with no baseline connection as potentially disastrous.

### Logistics

When selecting which meetings and congresses to attend, practical logistics are often important. Learners consider the day of the week, time of day, and how easy it is to reach the venue and return home. Some learners are reluctant to accept invitations to weekend events that interrupt time with family. Relevance to individual scope of practice is also considered, with some learners willing to make more of an effort to attend events focused on sub-specialist topics.

### Invitation clarity

Given the importance of topic relevance, learners may benefit from greater clarity around learning objectives and target audience in programme invitations. One learner remarked that she often cannot tell whether programmes are targeted for GPs, nurses, or specialists; having received the invitation is not strong enough of a message that she is among the intended target audience. Learners value efficiency and do not want to waste time on a programme that falls outside of their scope of practice.

### Interactivity and e-learning

When participating in e-learning and online activities, learners value interactivity as a tool for holding their attention. As one panellist remarked, “when we are prompted to answer questions, look at diagnostic images, or watch a video, we are interacting. We are not just sitting there for 15 minutes.” Learners agreed that questions embedded throughout the activity are helpful for staying engaged and tracking what has been learned. One panellist particularly appreciated what she described as “fear” questions (“uh oh, I don't know the answer. I'd better study this”). Post-activity questions can give learners a sense of accomplishment that they have indeed learned something new.

The ideal duration for an e-learning activity is 10–15 minutes, and nothing more than 30 minutes. One meeting delegate shared that an analysis of IP data from more than 700 learners showed that some completed a 30-minute module in only 5 minutes, while others had the activity open for 24 hours. In response, one of the panellists admitted that he is more likely to “cheat” when participating in a mandatory module, either by skipping ahead to the post-activity test (“remember, we are good at taking tests”), or leaving the activity open while he steps away to make some tea.

When asked what criteria they use to assess e-learning quality, the panellists admitted that they did not have specific criteria, but generally trusted the quality of activities distributed by medical societies over those developed by third parties. Despite the glut of online offerings, one learner shared that he did not know where to look to find e-learning programmes.

### Needs assessments

Learners are completely unfamiliar with the term “needs assessment” as it relates to CME–CPD. Rarely are they asked what they want to learn. Learners feel that selecting among concurrent sessions at large medical congresses is one of the few opportunities they have to drive their own learning.

### Value of accreditation

Surprisingly, the panellists were not able to differentiate between examples of accredited CME–CPD programmes and branded medical education. Furthermore, they did not appear to appreciate the differences between educational providers and accrediting bodies. For instance, learners perceived programmes held at universities or medical congresses as having more credibility than stand-alone activities, even though these activities may be accredited by the same accreditor.

### Industry funding

Although learners had concerns around bias related to industry support, they did not appear to understand when and where industry funding was at play in medical education. For example, learners reported that they “trusted the motivations” of national societies (i.e. CME–CPD events held in conjunction with national society meetings that may in fact be supported by industry funding), but questioned the motivations behind other types of programmes.

The overriding message is that learners do not understand how independent CME–CPD differs from branded medical education. Specific to CME–CPD programmes, learners are not aware of the differences between who provides the education, who accredits it, who funds it, and why these nuances matter.

## Session 7: the value of CME 3: what the future holds

In the third “Value of CME” session, Jacqui Thornton moderated the discussion among CME–CPD experts who reflected on the key take-away messages of the Lunch with the Learners session and offered their perspectives on the immediate future for CME. The multidisciplinary panel included Marie-Claire Pickaert (EFPIA), Edwin Borman (UEMS), Craig Campbell (RCPSC), and Peter Mills (Barts Heart Centre, European Cardiology Section Foundation Board Member).

### Trends in e-Learning

Keeping in mind that the term “e-learning” describes a spectrum of activity formats and educational designs, several general trends are emerging. First, the gap between high-quality activities and “unfortunate drivel” is growing. While some providers are developing innovative programmes that incorporate the latest evidence in adult learning principles, gaming theory, and other learning science, other providers continue to upload recordings of live lectures and call it a day. Unfortunately, some learners are not able to tell the difference and continue to consume poor-quality content.

On the plus side, there is increasing recognition for the need to cater e-learning activities to the individual needs of each learner. The technology that drives personalised content is ubiquitous, as evidenced by Amazon shopping suggestions and Netflix playlists. Providers need to apply that technology to better understand the educational needs and preferred learning styles of learners, and then supply personalised activity content. Embedded self-assessment questions can be used to provide continual customisation throughout the activity, with remedial explanations and supplemental content for learners who require more foundational knowledge.

Although some attendees expressed concerns about privacy issues, most agreed that the era of anonymous learner feedback is over. As one delegate said, “shame on us for not tracking them sooner,” to allow for better personalisation now. The next generation of learners will demand technology-driven learning opportunities. To maximize the potential of this format, providers must stay up-to-date on new e-learning platforms and options for data analysis.

Meeting attendees debated the role of accreditors in setting minimum quality standards for e-learning activities. Some argued that accreditors bear responsibility for preventing poor-quality e-learning activities from being unleashed on learners. However, to what extent should accreditation criteria be tied to the activity format? Accreditors need to hold providers accountable to meeting quality standards regardless of activity format. Moreover, there must be a strong, evidence-based rationale for the choice of format (“no more lectures unless there is evidence demonstrating that a lecture is the best tool for achieving a specific educational goal”).

### Raising awareness about CME–CPD

Learners appear to know little about what distinguishes independent accredited CME programs from branded promotional education, as demonstrated by feedback during the Lunch with the Learners session. It was acknowledged that the line between these types of education is getting fuzzier as industry-directed education begins to incorporate adult learning principles. Providers must do a better job at defining the value of CME, outlining the characteristics of accredited education, and earning learners’ trust as partners in their professional development.

There is also a need to get the target audience more invested in lifelong learning. CME–CPD providers can facilitate this by differentiating between education for trainees and education for consultants, who consider themselves to be fully trained. How do you get consultants to recognise they have compelling educational needs, and to articulate those needs? One panellist suggested posting the following question: Do you think you can maintain the necessary knowledge base to sustain the quality of your practice over the next 5 years without learning?

Healthcare professionals undertake a considerable amount of informal learning in the workplace. One meeting attendee estimated that informal workplace learning is the dominant source of education for many clinicians. Yet, employers representing hospitals, medical centres, and other environments where clinicians practise are overlooked as stakeholders in CME–CPD. Given the role of CME–CPD in performance improvement, employers should be part of the conversation. Moreover, workplace involvement in CME–CPD should support the goals of IPE, where clinicians who work together and deliver care together also learn together.

## Session 8: CME unsession

In the final #8ECF session, Lawrence Sherman guided delegates through a discussion designed to unravel the lessons learned and uncover any final questions. Meeting attendees agreed that one of the key take-home lessons involves the observation that many European healthcare professionals are fulfilling their CME–CPD requirements by attending one to two large annual medical society meetings. With rare exception, large medical congresses rarely address individual educational needs, nor address key topics such as IPE, collaboration, and other professional skills. Additional questions to ponder include: Who should take responsibility for educating healthcare professionals about what CME–CPD is? What is the best way to educate them? In the era of IPE, what does effective workplace learning look like?

## Summary

The 8th Annual European CME Forum was held at a time of growing focus on the quality and value of European CME. Approximately 100 participants gathered from Europe and beyond to share experiences and perspectives on key themes affecting the planning, development, delivery, and assessment of CME–CPD. European accreditors are evolving, with UEMS–EACCME considering updating their standards, EBAC addressing the challenges of identifying and reporting financial relationships, and national accreditation systems seeking to clarify their roles in their own countries. In 2016, industry is entering a new era of transparency and disclosure, with potential downstream effects on funding models, accountability standards, and engagement with the professional provider community. Remarking on whose responsibility it is to shape the future of European CME, one meeting delegate issued a call to action for fellow attendees: “We are all sitting around, holding our breath, turning blue, waiting for somebody else to tell us how to do this. People in this room need to claim that authority and do it.” In effect, all stakeholders must advocate for the highest quality standards across the spectrum of CME–CPD. No doubt progress towards that goal, and much more, will be on the agenda at the 9th Annual European CME Forum (#9ECF), which will take place in Amsterdam, The Netherlands, on 9–11 November 2016.

## Conflict of interest and funding

The authors have not received any funding or benefits from industry or elsewhere to generate and submit this report.

## References

[1] Alliance for Continuing Education in the Health Professions (ACEhP) 40th Annual Conference, Grapevine, Texas, 14–17 January 2015. Available at: http://www.eventscribe.com/2015/acehpannual/, accessed May 16, 2016.

[2] Good CME Practice group (gCMEp). Available at: http://www.goodcmepractice.eu, accessed May 16, 2016.

[3] European Board for Accreditation in Cardiology (EBAC) Available at: http://www.ebac-cme.org, accessed May 16, 2016. 10.3402/jecme.v5.29757 PMC5843057 29644119

[4] Accreditation Council for CME (ACCME) Accreditation Council for CME names Graham T. McMahon, MD, MMSc, as new President and CEO beginning April 2015. 12 January 2015. Available at: http://www.accme.org/news-publications/news, accessed May 16, 2016.

[5] Srivastava V, Sullivan L, Sanghvi S CME/CPD in the Indian Subcontinent: proceedings from the 1st regional meeting of Global Alliance for Medical Education (GAME) in Mumbai, India. J Eur CME. 2015; 4 27499, doi: http://dx.doi.org/10.3402/jecme.v4.27499.

[6] Simper J Cologne Consensus Conference: providers in accredited CME/CPD 11–12 September 2015, Cologne, Germany. J Eur CME. 2016; 5 31437, doi: http://dx.doi.org/10.3402/jecme.v5.31437. 10.3402/jecme.v5.31437 PMC5843054 29644122

[7] Stevenson R, Olson CA Collaboration between JECME and JCEHP: providing new opportunities for CME researchers and practitioners. J Contin Educ Health Prof. 2015; 35: 157. 26378420 10.1002/chp.21297

[8] UEMS-EACCME Report of the Secretary General UEMS Council Meeting, Warsaw, Poland, 16–17 October 2015. Available at: http://www.uems.eu/__data/assets/pdf_file/0004/27832/3.-UEMS-2015_26-Report-of-the-UEMS-Secretary-General-FINAL.pdf, accessed May 16, 2016..

[9] Farrow S, Gillgrass D, Pearlstone A, Torr J, Pozniak E, Good CME Practice Group Setting CME standards in Europe: guiding principles for medical education. Curr Med Res Opin. 2012; 28: 1861–1871. [PubMed Abstract]. 23043468 10.1185/03007995.2012.738191

[10] Wiggins G, McTighe J Understanding by design (Expanded 2nd ed.). 2005; Alexandria, VA: Association for Supervision and Curriculum Development.

[11] Moore DE Jr., Green JS, Gallis HA Achieving desired results and improved outcomes: integrating planning and assessment throughout learning activities. J Contin Educ Health Prof. 2009; 29: 1–15. 19288562 10.1002/chp.20001

[12] Accreditation Council for CME (ACCME) ACCME opens accreditation process to international CME providers. 2014 Available at: http://www.accme.org/news-publications/highlights/accme-opens-accreditation-process-international-cme-providers, accessed May 16, 2016.

[13] National Institute for Health and Care Excellence (NICE) Rheumatoid arthritis in adults: management. NICE guidelines [CG79]. 2015 Available at: www.nice.org.uk/guidance/cg79, accessed May 16, 2016. 30102507

[14] National Institute for Health and Care Excellence (NICE) Adalimumab, etanercept, infliximab, certolizumab pegol, golimumab, tocilizumab and abatacept for rheumatoid arthritis not previously treated with DMARDs or after conventional DMARDs only have failed. NICE technology appraisal guidance [TA375]. 2016 Available at: http://www.nice.org.uk/guidance/ta375, accessed May 16, 2016.

[15] Griebenow R, Campbell C, Qaseem A, Hayes S, Gordon J, Michalis L Proposal for a graded approach to disclosure of interests in accredited CME/CPD. J Eur CME. 2015; 4 29894, doi: http://dx.doi.org/10.3402/jecme.v4.29894.

[16] General Medical Council Promoting excellence: standards for medical education and training. 2015 Available at: http://www.gmc-uk.org/education/standards.asp, accessed May 16, 2016.

